# Work Experience of Sessional Teaching Staff in Pre‐Registration Nursing Programmes: A Cross‐Sectional Study

**DOI:** 10.1002/nop2.70862

**Published:** 2026-09-29

**Authors:** Thomas Aaron Ricks, Elisabeth Jacob, Louisa Lam

**Affiliations:** ^1^ School of Nursing, Midwifery & Paramedicine Australian Catholic University Fitzroy Victoria Australia; ^2^ Nurse Workforce Unit St Vincent's Hospital Melbourne Fitzroy Victoria Australia; ^3^ Lifestyle Medicine & Health Research Centre Avondale University Cooranbong New South Wales Australia; ^4^ School of Public Health Monash University Melbourne Victoria Australia

**Keywords:** casual, experience, nursing education, psychosocial factors, sessional, sustainability, workforce

## Abstract

**Background:**

Internationally, sessional nursing staff play an important role in meeting the teaching requirements for the increased demand in nursing student enrolment. In Australia, pre‐registration nursing programmes are university programmes that prepare students for initial registration as registered nurses. Little research has been undertaken to understand their experiences to ensure a sustainable nurse education workforce.

**Objective:**

To explore the experiences of sessional staff working in pre‐registration nursing programmes in Australia.

**Design:**

A cross‐sectional online survey.

**Setting and Participants:**

Sessional nursing staff working in pre‐registration nursing programmes in Australia for at least 12 months.

**Methods:**

Participants were invited to complete an online survey using the Copenhagen Psychosocial Questionnaire version III (COPSOQ–III) middle‐length version. Participants were recruited via email and online platforms. Data were analysed using descriptive and non‐parametric techniques including determining correlations between different subscales.

**Results:**

A total of 84 participants were included. Most participants were female (83.3%) and over 50 years old (51.2%). The majority of the participants had less than 5 years of sessional experience (58.3%). Only 26.2% (*n* = 22) of the participants intended to become permanent academics. Participants generally reported favourable working experiences. ‘Job satisfaction’ was found to be positively correlated with several subscales including ‘social support from supervisor’ (*r* = 0.64, *p* < 0.001), ‘social support from colleagues’ (*r* = 0.54, *p* < 0.001) and ‘sense of community’ (*r* = 0.64, *p* < 0.001).

**Conclusion:**

This study provides new insights into the sessional nursing workforce, including their demographics and work experiences. It identified factors related to positive work experience, such as opportunities for professional development, sense of community, quality leadership and support. Strengthening these factors may improve the work experience of sessional staff, contribute to reduced attrition and support workforce sustainability.

**Abbreviations**
AHPRA: Australian Health Practitioner Regulation Agency
COPSOQ‐III: Copenhagen Psychosocial Questionnaire version III
EQUATOR: Enhancing the Quality and Transparency of health research
IBM: International Business Machine
IP: internet protocol
QR: quick response
SD: standard deviation
SPSS: statistical package for the social sciences
STROBE: Strengthening the Reporting of Observational Studies in Epidemiology
UK: United Kingdom
USA: United States of America

## 1 Introduction

The nursing workforce shortage is an issue worldwide and there is a projected deficit of nearly 4.5 million nurses by the year 2030 (Boniol et al. 2022). This shortage is caused by various aspects including human resource management issues, the ageing of the academic workforce and population worldwide, personal factors such as nurses' motivations and external factors such as poor working conditions in healthcare settings (Tamata and Mohammadnezhad 2022; World Health Organization 2025a). Some of the human resource management issues include poor policy and planning for increasing nursing numbers, inadequate recruitment and a lack of leadership and management in the nursing workforce (Alreshidi et al. 2022; Marć et al. 2019; Marufu et al. 2021; Park and Yu 2019). In addition, many nurses who are already in the profession intend to leave clinical practice due to factors such as poor clinical working conditions, unattractive working environments and low salaries (Kakemam et al. 2019; Marć et al. 2019; Marufu et al. 2021; Sirisub et al. 2019). Organisations such as the World Health Organization (2025b) and the International Council of Nurses (2021) have called for strategies to address the nursing workforce shortage, including expanding access to nurse education, strengthening recruitment into the profession and improving retention of the existing workforce. This has led to more individuals pursuing a nursing career (Tamata and Mohammadnezhad 2022). As a result, there is an increased demand for nurses to enter the workforce which has increased nursing student enrolment (Fangonil‐Gagalanga et al. 2023; Petges and Sabio 2020; World Health Organization 2025b). The growth in the number of nursing students internationally has led universities to require more sessional nursing teaching staff to address this challenge (Jarosinski et al. 2022).

There are many reasons why nurses want to be part of the sessional teaching workforce including that sessional teaching work provides enjoyment and flexibility in work arrangements. Staff are generally motivated to teach by the opportunity to share knowledge, influence the future generation workforce, develop positive relationships with students and facilitate learning activities (Frenzel et al. 2018; Xie et al. 2023). Sessional teaching staff feel privileged to provide their nursing expertise and knowledge from their clinical practice as they can positively influence the next generation of nurses (Dixon et al. 2015; McComb and Eather 2024; Ricks et al. 2025; Woodworth 2016). Other reasons include the ability to choose the type of work they undertake (teaching, clinical facilitation or marking), greater control over when they work, and improved work‐life balance (Amer et al. 2022; Aust et al. 2023). From a university perspective, employing sessional teaching staff can reduce expenditures including superannuation and leave entitlements such as annual or parental leave (Bodak et al. 2019; Fair Work Ombudsman Australian Government, n.d.; Heffernan 2018). This has led to casualisation in the higher education sector including the nurse education workforce (Bodak et al. 2019; Brouwer et al. 2024; Dixon et al. 2015; McComb and Eather 2024; Williams 2021).

Internationally, there is a high proportion of sessional staff teaching in the higher education sector including Australia, New Zealand, the United States of America (USA), the United Kingdom (UK) and some European Union countries (Brouwer et al. 2024; Croucher 2023; Harvey 2017; Hattam and Weiler 2022; Leathwood and Read 2022; McComb et al. 2021; McComb and Eather 2024; Stubaus 2015). In Australia, nearly 60% of the workforce is made up of non‐permanent academics (McComb and Eather 2024). In the USA, nearly 70% of academics are sessional staff and in the UK 54% are non‐continuing teaching staff (Culver and Kezar 2021; Leathwood and Read 2022). Similarly, in Latvia, sessional staff accounted for 78% of academics in higher education in 2022 (Rusakova and Baranova 2021). Given the substantial use of sessional staff in nurse education internationally, it is important to understand their working experiences including job satisfaction, sense of community, access to support and opportunities for academic development. These factors are central to supporting the sustainability of the sessional academic workforce (Ricks et al. 2025). However, existing evidence on sessional nursing academics remains limited and fragmented, with previous research providing little national quantitative evidence about their psychosocial working conditions, including job satisfaction. Research has more commonly examined permanent nursing academics or broader groups of sessional academics, limiting understanding of the distinct experiences of sessional staff working specifically within pre‐registration nursing education (East et al. 2024; Ricks et al. 2025; Singh et al. 2021). Addressing this gap is important for developing evidence‐informed strategies to support and sustain this workforce. For the purpose of this study, sessional or casual teaching staff in nurse education are those who are not employed permanently or do not have a fixed‐term or continuing contract with universities and are employed to perform duties such as direct teaching, marking or clinical placement facilitation (Ricks et al. 2025).

## 2 Materials and Methods

### 2.1 Aim

This study aimed to explore the experience of sessional nursing staff working in pre‐registration nurse education programmes in Australia.

### 2.2 Study Design

A quantitative cross‐sectional study design was used for this study. The survey was administered via an online platform, Qualtrics (https://www.qualtrics.com). This study adhered to the Strengthening the Reporting of Observational Studies in Epidemiology (STROBE) reporting guideline for cross‐sectional studies from the Enhancing the QUAlity and Transparency Of health Research (EQUATOR) network (Vandenbroucke et al. 2007).

### 2.3 Settings

The study was conducted in August 2024 in Australia across multiple universities.

### 2.4 Participants

Participants included sessional nursing staff working in Australian pre‐registration nurse education programmes, which prepare students for initial registration as registered nurses. The inclusion criteria were: (1) Registered Nurse with the Australian Health Practitioner Regulation Agency (AHPRA), (2) working as a sessional staff member in a pre‐registration nursing program, (3) having at least 12 months of working experience as a sessional staff member and (4) working in Australia. Participants with only postgraduate nursing teaching experience were not eligible for this study.

### 2.5 Participant Selection

A convenience sampling method was used to recruit participants in Australia. This approach was selected because no national sampling frame or comprehensive register of sessional nursing academics in Australia was available, and it enabled recruitment across multiple institutions and professional networks nationally. We emailed the study information sheet and survey links, which included a quick response (QR) code to the survey, to the heads of discipline in pre‐registration nursing programmes at 34 Australian educational institutions in August 2024. The eligible pre‐registration nursing programmes in Australia were first identified through the approved study programmes for registered nurses on the AHPRA website (Australian Health Practitioner Regulation Agency, n.d.). The contact details of the heads of discipline in nursing were identified through their educational institution's websites or administration staff. The survey remained open for 1 month during August 2024. We also used other advertising methods including posting a flyer on The Buzz—Australian College of Nursing and posting the advertisement on the social media platform LinkedIn to increase the participation rate.

### 2.6 Data Collection Instrument

The survey collected participants' demographic data (e.g., age, sex, years of experience as a sessional staff member, and the highest level of qualification obtained) and variables related to their working experiences as sessional nursing staff using the middle‐length version of the Copenhagen Psychosocial Questionnaire version III (COPSOQ–III) (https://www.copsoq‐network.org) (Llorens et al. 2019). The COPSOQ–III is a workplace psychosocial risk assessment instrument designed to collect psychosocial and health‐related data to understand workplace experience. The data allow organisations to benchmark risk factors to understand employees' health and well‐being. The middle‐length version was chosen to reduce participant burden and minimise the risk of non‐response or incomplete survey responses. This version of the instrument consists of 26 subscales comprising 60 items with each subscale containing at least one question and up to four questions. All items use a five‐point Likert scale, with numerical values allocated for analysis (e.g., To a very large extent = 100, To a large extent = 75, Somewhat = 50, To a small extent = 25, To a very small extent = 0). The numerical values assigned to each Likert scale rating were specified in the COPSOQ–III instructions for data analysis purposes. The middle‐length version of the COPSOQ–III does not include any reverse‐scored items. The survey contained questions related to different subscales including ‘job satisfaction’, ‘sense of community’, ‘social support from supervisor’, ‘social support from colleagues’, ‘meaning of work’, ‘horizontal trust’, ‘vertical trust’, ‘recognition’, ‘job insecurity’, ‘insecurity over working conditions’ (see Table 1).

**TABLE 1:** COPSOQ–III subscales and question items.

Subscale and abbreviation	Question items
Quantitative demands (QD)^a^	Is your workload unevenly distributed so it piles up? How often do you not have time to complete all your work tasks? Do you get behind with your work?
Work pace (WP)^a^, ^b^	Do you have to work very fast? Do you work at a high pace throughout the day?
Emotional demands (ED)^a^, ^b^	Does your work put you in emotionally disturbing situations? Do you have to deal with other people's personal problems as part of your work? Is your work emotionally demanding?
Demands for hiding emotions (HE)^a^, ^b^	Does your work require that you hide your feelings? Are you required to be kind and open towards everyone – regardless of how they behave towards you? Does your work require that you do not state your opinion?
Influence at work (IN)^a^	Do you have a large degree of influence on the decisions concerning your work? Can you influence the amount of work assigned to you? Do you have any influence on what you do at work? Do you have any influence on HOW you do your work?
Possibilities for development (PD)^b^	Do you have the possibility of learning new things through your work? Can you use your skills or expertise in your work? Does your work give you the opportunity to develop your skills?
Control over working time (CT)^a^	Can you decide when to take a break? Can you take holidays more or less when you wish? Can you leave your work to have a chat with a colleague? If you have some private business is it possible for you to leave your place of work for half an hour without special permission?
Meaning of work (MW)^b^	Is your work meaningful? Do you feel that the work you do is important?
Predictability (PR)^b^	At your place of work, are you informed well in advance concerning for example important decisions, changes or plans for the future? Do you receive all the information you need in order to do your work well?
Recognition (RE)^b^	Is your work recognised and appreciated by the management?
Role clarity (CL)^b^	Does your work have clear objectives? Do you know exactly which areas are your responsibility? Do you know exactly what is expected of you at work?
Role conflicts (CO)^b^	Are contradictory demands placed on you at work? Do you sometimes have to do things which ought to have been done in a different way?
Illegitimate tasks (IT)^b^	Do you sometimes have to do things which seem to be unnecessary?
Quality of leadership (QL)^b^	To what extent would you say that your immediate superior … makes sure that the members of staff have good development opportunities? is good at work planning? is good at solving conflicts?
Social support from supervisor (SS)^a^	How often is your immediate superior willing to listen to your problems at work, if needed? How often do you get help and support from your immediate superior, if needed?
Social support from colleagues (SC)^a^	How often do you get help and support from your colleagues, if needed? How often are your colleagues willing to listen to your problems at work, if needed?
Sense of community at work (SW)^a^	Is there a good atmosphere between you and your colleagues? Do you feel part of a community at your place of work?
Job insecurity (JI)^b^	Are you worried about becoming unemployed? Are you worried about it being difficult for you to find another job if you became unemployed?
Insecurity over working conditions (IW)^b^	Are you worried about being transferred to another job against your will? Are you worried about the timetable being changed (shift, weekdays, time to enter and leave …) against your will? Are you worried about a decrease in your salary (reduction, variable pay being introduced …)?
Quality of work (QW)^b^	Are you satisfied with the quality of the work performed at your workplace?
Job satisfaction (JS)^c^	Regarding your work in general. How pleased are you with your work prospects? your job as a whole, everything taken into consideration? your salary?
Work life conflict (WF)^a^	The next two questions concern the ways in which your work affects your private life: Do you feel that your work drains so much of your energy that it has a negative effect on your private life? Do you feel that your work takes so much of your time that it has a negative effect on your private life?
Horizontal trust (TE)^b^	Do the employees in general trust each other?
Vertical trust (TM)^b^	Does the management trust the employees to do their work well? Can the employees trust the information that comes from the management? Are the employees able to express their views and feelings?
Organisational justice (JU)^b^	Are conflicts resolved in a fair way? Is the work distributed fairly?
Self Rated Health (GH)^d^	In general, would you say your health is

^a^ Likert scales: Always (100); Often (75); Sometimes (50); Seldom (25); Never/hardly ever (0), Missing/No supervisors/colleagues (coded as missing).

^b^ To a very large extent (100); To a large extent (75); Somewhat (50); To a small extent (25); To a very small extent (0).

^c^ Very satisfied (100); Satisfied (75); Neither/Nor (50); Unsatisfied (25); Very unsatisfied (0).

^d^ Excellent (100); Very good (75); Good (50); Fair (25); Poor (0).

### 2.7 Data Collection Procedure

After accessing the survey through the link or QR code, participants were first presented with the study information and consent form. Participants indicated their consent by selecting the acceptance option in Qualtrics. Those who provided consent proceeded to the eligibility questions. Eligible participants then completed demographic and sessional employment questions, followed by the 60 COPSOQ–III items. The median survey completion time among participants included in the analysis was approximately 12 min.

### 2.8 Data Analysis

No formal a priori sample‐size calculation was undertaken because no national sampling frame or reliable estimate of the size of the sessional nursing academic workforce in Australia was available. Recruitment was therefore based on feasibility with the aim of maximising the number of eligible responses obtained nationally during the recruitment period. The analyses should be interpreted as exploratory.

Data analysis involved descriptive statistics and non‐parametric tests. Data analysis was conducted using the International Business Machines Statistical Package for the Social Sciences (IBM SPSS) version 30.0. Before analysis, responses were screened for eligibility and completeness. Responses from individuals who did not meet the eligibility criteria or complete the eligibility questions were excluded. Missing responses were coded as missing in SPSS and were not imputed. Eligible responses were retained where sufficient data were available for the relevant analysis. For each participant, a multi‐item subscale score was calculated by averaging the completed items, provided that at least half of the items in that subscale had been answered. If fewer than half of the items had been answered, the participant's subscale score was treated as missing.

Mean scores were calculated for each COPSOQ–III subscale, with multi‐item subscale scores calculated from their constituent items as recommended by Ose et al. (2023). Spearman's rank correlation coefficients (*r*) were selected because the survey used Likert‐scale responses that produced ordinal data and were not assumed to be normally distributed. As a non‐parametric method, Spearman's correlation did not require the assumption of normally distributed data. Spearman's rank correlation coefficients (*r*) were used to estimate associations between subscales, and Fisher's r‐to‐z transformation was used to calculate 95% confidence intervals (Bishara and Hittner 2017). Statistical significance was set at *p* < 0.05, and correlation coefficients were interpreted according to Pallant (2020) guidelines for the strength of association. No adjustment for multiple testing was applied because the analyses were exploratory rather than confirmatory.

### 2.9 Data Validity

Internal consistency was assessed using Cronbach's alpha (*α*) for COPSOQ–III subscales containing more than one item. According to the interpretive guidance described by Taber (2018), Cronbach's alpha values indicated variable internal consistency across the subscales, ranging from 0.564 to 0.922, with the exception of the ‘work pace’ subscale. Several subscales had Cronbach's alpha values below 0.70, including ‘quantitative demands’ (*α* = 0.564), ‘possibilities for development’ (*α* = 0.637), ‘social support from supervisor’ (*α* = 0.656), ‘job satisfaction’ (*α* = 0.664) and ‘predictability’ (*α* = 0.692). These values indicate modest internal consistency and suggest that findings involving these subscales should be interpreted cautiously. However, the subscales were retained because they were established COPSOQ–III constructs, contained a small number of items and were used within an exploratory analysis. Cronbach's alpha could not be calculated for single‐item subscales. The ‘work pace’ subscale demonstrated low internal consistency (*α* = 0.285). This indicated that the two items did not form a sufficiently internally consistent subscale in this sample; consequently, the subscale was excluded from further analysis, reducing the study's ability to evaluate participants’ work pace. This limitation was specific to the ‘work pace’ subscale, although the broader variation in Cronbach's alpha values indicates that reliability was not uniform across the COPSOQ–III subscales in this sample and should be considered when interpreting the findings (see Supplementary File S1).

### 2.10 Ethical Considerations

This study was approved by the Australian Catholic University Human Research Ethics Committee (HREC) (Reference: 2024‐3120E). All participants were provided with the study information and consent form at the commencement of the survey. Apart from the eligibility questions, all survey questions were voluntary. Data were collected anonymously without tracking the Internet Protocol (IP) address.

## 3 Results

A total of 99 survey responses were received. Following eligibility screening, 15 responses were excluded: four because participants had less than 12 months of sessional experience, one because the participant was not a registered nurse, and 10 because the eligibility questions were not completed. Therefore, 84 participants were included in the analysis (see Figure 1). Participants reported employment across 25 of the 34 educational institutions contacted, representing institutional coverage of 73.5%.

**FIGURE 1:**
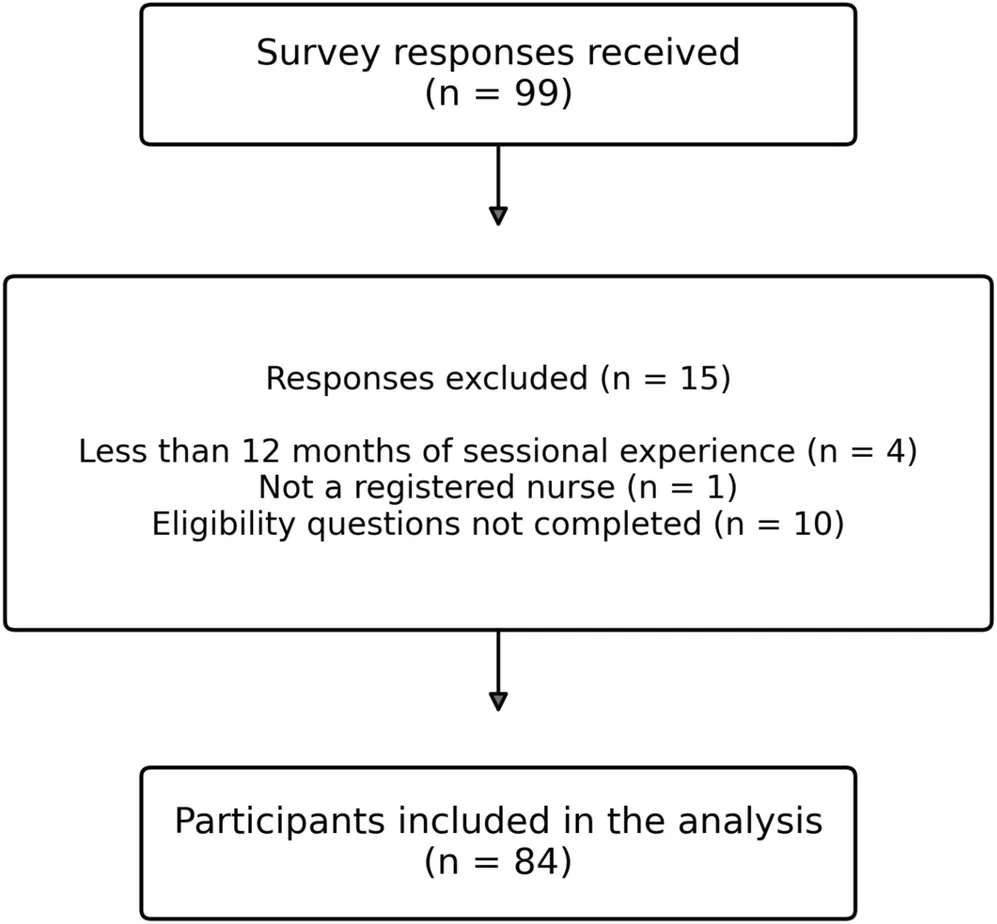
Flow diagram of survey responses, exclusions and participants included in the analysis.

### 3.1 Demographics

Participants represented 25 Australian educational institutions and demonstrated variation in age, qualifications, professional experience, years of sessional employment, working arrangements and intention to pursue permanent academic employment. However, the representativeness of the sample relative to the broader Australian sessional nursing academic workforce could not be determined because no national workforce profile or sampling frame was available.

Most participants were over 50 years old (*n* = 43; 51.2%) and female (*n* = 70; 83.3%). The largest proportion of participants reported their highest qualification as a graduate certificate, graduate diploma or honours degree (*n* = 46; 54.8%). Over 77% of participants (*n* = 65) were Australian‐educated registered nurses while a small proportion held additional registration in midwifery and/or paramedicine (*n* = 10; 11.9%). Participants generally had over 10 years of working experience as registered health professionals (*n* = 75; 89.2%), although most had less than 5 years of working experience as sessional staff in university education (*n* = 49; 58.3%). The majority of the participants spent less than 20 h per week working during a semester (*n* = 67; 79.8%). Most participants were currently working clinically (*n* = 54; 64.3%), and most had carer responsibilities (*n* = 60; 71.4%). Interestingly, only 26.2% (*n* = 22) of participants intended to seek permanent academic positions. A small percentage of the participants (*n* = 16; 19%) worked only off‐campus whereas most participants worked either on‐campus (*n* = 28; 33.3%) or a combination of on and off‐campus (*n* = 36; 42.9%) (see Table 2). No correlations were found between demographics and the COPSOQ–III subscales.

**TABLE 2:** Demographic and professional characteristics of participants (*N* = 84).

	*n* (Total 84)	%
Age		
18–30	3	3.6
31–40	6	7.1
41–50	14	16.7
51–60	21	25.0
Over 60	22	26.2
Missing/Prefer not to say	18	21.4
Sex		
Male	10	11.9
Female	70	83.3
Missing/Prefer not to say	4	4.8
Highest level of qualification		
Below undergraduate	1	1.2
Undergraduate	1	1.2
Graduate Certificate/Graduate Diploma/Honours	46	54.8
Master	21	25.0
PhD or Doctoral	11	13.1
Missing/Prefer not to say	4	4.8
Country of initial nursing training		
In Australia	65	77.4
Overseas	15	17.9
Missing/Prefer not to say	4	4.8
Registration status		
Registered Nurse only	70	83.3
Registered Nurse and Registered Midwife	7	8.3
Registered Nurse and Registered Paramedic	2	2.4
Registered Nurse, Registered Midwife and Registered Paramedic	1	1.2
Missing/Prefer not to say	4	4.8
Years of experience as a health professional		
Less than 5 years	3	3.6
5–10 years	2	2.4
More than 10 years	75	89.2
Missing/Prefer not to say	4	4.8
Years of experience as sessional		
Less than 5 years	49	58.3
5 to 10 years	28	33.3
More than 10 years	7	8.3
Average hours of work per week during a semester		
Less than 5 h/week	26	31.0
5–10 h/week	27	32.1
10–20 h/week	14	16.7
20–30 h/week	9	10.7
More than 30 h/week	4	4.8
Missing/Prefer not to say	4	4.8
Intend to become permanent academic		
Yes	22	26.2
No	23	27.4
Maybe	34	40.5
Missing/Prefer not to say	5	6.0
Work on campus		
Yes	28	33.3
No	16	19.0
Both on and off campus	36	42.9
Missing/Prefer not to say	4	4.8
Currently work clinically		
Yes	54	64.3
No	25	29.8
Missing/Prefer not to say	5	6.0
Have carer responsibility		
Yes	60	71.4
No	19	22.6
Missing/Prefer not to say	5	6.0
Have home life that may impact ability to work sessionally		
Yes	27	32.1
No	49	58.3
Missing/Prefer not to say	8	9.5

*Note:* Percentages were calculated using the total sample (*N* = 84) with ‘Missing/Prefer not to say’ responses included in the denominator. Percentages may not total 100% because of rounding.

### 3.2 Working Experience

The COPSOQ–III subscales were interpreted according to the direction of the construct being measured. Resource‐related subscales reflect supportive organisational, relational and motivational aspects of work, where higher mean scores indicate more favourable working experiences. In contrast, demand‐related subscales reflect aspects of work that may contribute to strain, conflict, or insecurity, where lower mean scores indicate more favourable working experiences.

Participants reported favourable scores across several resource‐related subscales, with mean scores above 60 observed for 10 subscales (see Table 3). These included ‘meaning of work’ (mean = 79.61), ‘role clarity’ (mean = 74.66), ‘social support from supervisor’ (mean = 64.77), ‘social support from colleagues’ (mean = 61.69), ‘job satisfaction’ (mean = 61.85), ‘vertical trust’ (mean = 61.03), ‘horizontal trust’ (mean = 60.87), ‘quality of work’ (mean = 60.71), ‘possibilities for development’ (mean = 60.64) and ‘sense of community at work’ (mean = 60.04). These findings suggest that participants generally reported favourable organisational resources, particularly in relation to meaningful work, role clarity, support, trust and collegial connection.

**TABLE 3:** Mean (SD) scores of COPSOQ–III subscales (*N* = 84).

Subscales	Mean (SD)
Quantitative demands (QD)	43.57 (19.24)
Emotional demands (ED)	30.15 (21.94)
Demands for hiding emotions (HE)	45.07 (21.87)
Influence at work (IN)	46.71 (23.01)
Possibilities for development (PD)	60.64 (18.24)
Control over working time (CT)	46.13 (28.85)
Meaning of work (MW)	79.61 (21.10)
Predictability (PR)	55.26 (24.09)
Recognition (RE)	47.04 (30.81)
Role clarity (CL)	74.66 (19.43)
Role conflicts (CO)	32.94 (25.81)
Illegitimate tasks (IT)	34.21 (26.09)
Quality of leadership (QL)	53.59 (24.46)
Social support from supervisor (SS)	64.77 (28.24)
Social support from colleagues (SC)	61.69 (28.80)
Sense of community at work (SW)	60.04 (29.60)
Job insecurity (JI)	32.57 (34.94)
Insecurity over working conditions (IW)	22.07 (25.79)
Quality of work (QW)	60.71 (25.74)
Job satisfaction (JS)	61.85 (19.19)
Work life conflict (WF)	27.64 (26.80)
Horizontal trust (TE)	60.87 (23.67)
Vertical trust (TM)	61.03 (24.31)
Organisational justice (JU)	55.99 (24.08)
Self‐rated health (GH)	73.73 (20.76)

*Note:* COPSOQ–III subscale scores range from 0 to 100. For resource‐related subscales, higher scores indicate more favourable working experiences. For demand‐related subscales, lower scores indicate more favourable working experiences.

Abbreviation: SD, standard deviation.

Participants also reported favourable scores across several demand‐related subscales, with six subscales having mean scores below 40 (see Table 3). These included ‘insecurity over working conditions’ (mean = 22.07), ‘work‐life conflict’ (mean = 27.64), ‘emotional demands’ (mean = 30.15), ‘job insecurity’ (mean = 32.57), ‘role conflicts’ (mean = 32.94) and ‘illegitimate tasks’ (mean = 34.21). As lower scores on these subscales indicate more favourable outcomes, these findings suggest that participants reported relatively low levels of insecurity, work‐life conflict, emotional demands, role conflict and illegitimate tasks.

Correlations were estimated between all COPSOQ–III subscales. The following four positively oriented subscales were selected for detailed reporting because they showed statistically significant positive correlations with multiple other COPSOQ–III subscales and were conceptually relevant to participants' overall working experiences: ‘job satisfaction’, ‘sense of community at work’, ‘social support from supervisor’ and ‘social support from colleagues’. These correlations suggest that satisfaction, collegial connection and perceived support were closely related to broader psychosocial working conditions among sessional nursing academics.

### 3.3 ‘Job Satisfaction’ Correlations

The mean score for the ‘job satisfaction’ subscale was 61.85 out of 100 (SD = 19.19). This represents positive job satisfaction for the majority of sessional staff in our study. The psychosocial factors that had strong positive correlations with the ‘job satisfaction’ subscale were: ‘organisational justice’ (*r* = 0.82, *p* < 0.001), ‘vertical trust’ (*r* = 0.81, *p* < 0.001), ‘quality of leadership’ (*r* = 0.79, *p* < 0.001), ‘role clarity’ (*r* = 0.70, *p* < 0.001), ‘possibilities for development’ (*r* = 0.69, *p* < 0.001), ‘social support from supervisor’ (*r* = 0.64, *p* < 0.001), ‘sense of community at work’ (*r* = 0.64, *p* < 0.001), ‘meaning of work’ (*r* = 0.63, *p* < 0.001) and ‘predictability’ (*r* = 0.63, *p* < 0.001) (see Table 4).

**TABLE 4:** COPSOQ–III subscale correlations.

Subscales and abbreviations	Job satisfaction (JS)		Sense of community (SW)		Social support from supervisor (SS)		Social support from colleagues (SC)	
	*r* (95% CI^a^, ^b^)	*p*	*r* (95% CI^a^, ^b^)	*p*	*r* (95% CI^a^, ^b^)	*p*	*r* (95% CI^a^, ^b^)	*p*
Quantitative demands (QD)	0.35 (0.15–0.51)	< 0.001*	0.39 (0.20–0.55)	< 0.001*	0.32 (0.13–0.49)	0.001*	0.31 (0.12–0.48)	0.002*
Emotional demands (ED)	0.46 (0.28–0.60)	< 0.001*	0.38 (0.19–0.54)	< 0.001*	0.28 (0.09–0.46)	0.005*	0.30 (0.11–0.48)	0.002*
Demands for hiding emotions (HE)	0.41 (0.22–0.56)	< 0.001*	0.31 (0.12–0.48)	0.002*	0.29 (0.09–0.46)	0.004*	0.28 (0.08–0.46)	0.005*
Influence at work (IN)	0.52 (0.35–0.65)	< 0.001*	0.61 (0.46–0.72)	< 0.001*	0.61 (0.47–0.73)	< 0.001*	0.61 (0.46–0.72)	< 0.001*
Possibilities for development (PD)	0.69 (0.57–0.79)	< 0.001*	0.59 (0.44–0.71)	< 0.001*	0.62 (0.47–0.73)	< 0.001*	0.52 (0.35–0.65)	< 0.001*
Control over working time (CT)	0.51 (0.34–0.65)	< 0.001*	0.51 (0.34–0.64)	< 0.001*	0.55 (0.39–0.67)	< 0.001*	0.56 (0.40–0.68)	< 0.001*
Meaning of work (MW)	0.63 (0.48–0.74)	< 0.001*	0.57 (0.42–0.70)	< 0.001*	0.53 (0.37–0.66)	< 0.001*	0.44 (0.26–0.59)	< 0.001*
Predictability (PR)	0.63 (0.49–0.74)	< 0.001*	0.63 (0.49–0.74)	< 0.001*	0.55 (0.39–0.68)	< 0.001*	0.56 (0.40–0.69)	< 0.001*
Recognition (RE)	0.41 (0.19–0.58)	< 0.001*	0.33 (0.10–0.52)	0.004*	0.41 (0.20–0.59)	< 0.001*	0.35 (0.13–0.54)	0.002*
Role clarity (CL)	0.70 (0.58–0.79)	< 0.001*	0.60 (0.45–0.71)	< 0.001*	0.62 (0.48–0.73)	< 0.001*	0.59 (0.44–0.71)	< 0.001*
Role conflicts (CO)	0.46 (0.28–0.61)	< 0.001*	0.46 (0.28–0.60)	< 0.001*	0.42 (0.23–0.57)	< 0.001*	0.47 (0.29–0.61)	< 0.001*
Illegitimate tasks (IT)	−0.21 (−0.42–0.02)	0.069	−0.04 (−0.27–0.19)	0.717	−0.17 (−0.38–0.07)	0.151	−0.07 (−0.29–0.17)	0.573
Quality of leadership (QL)	0.79 (0.70–0.86)	< 0.001*	0.67 (0.55–0.77)	< 0.001*	0.73 (0.62–0.81)	< 0.001*	0.64 (0.50–0.75)	< 0.001*
Social support from supervisor (SS)	0.64 (0.51–0.75)	< 0.001*	0.70 (0.58–0.79)	< 0.001*	N/A	N/A	0.76 (0.66–0.83)	< 0.001*
Social support from colleagues (SC)	0.54 (0.38–0.67)	< 0.001*	0.77 (0.68–0.84)	< 0.001*	0.76 (0.66–0.83)	< 0.001*	N/A	N/A
Sense of community at work (SW)	0.64 (0.50–0.75)	< 0.001*	N/A	N/A	0.70 (0.58–0.79)	< 0.001*	0.77 (0.68–0.84)	< 0.001*
Job insecurity (JI)	0.49 (0.32–0.63)	< 0.001*	0.44 (0.26–0.59)	< 0.001*	0.44 (0.26–0.59)	< 0.001*	0.42 (0.24–0.58)	< 0.001*
Insecurity over working conditions (IW)	0.47 (0.30–0.61)	< 0.001*	0.45 (0.27–0.60)	< 0.001*	0.45 (0.27–0.60)	< 0.001*	0.42 (0.24–0.57)	< 0.001*
Quality of work (QW)	0.27 (0.03–0.48)	0.026*	0.09 (−0.16–0.32)	0.478	0.08 (−0.16–0.32)	0.498	−0.04 (−0.28–0.20)	0.731
Job satisfaction (JS)	N/A	N/A	0.64 (0.50–0.75)	< 0.001*	0.64 (0.51–0.75)	< 0.001*	0.54 (0.38–0.67)	< 0.001*
Work life conflict (WF)	0.44 (0.26–0.59)	< 0.001*	0.52 (0.36–0.66)	< 0.001*	0.49 (0.32–0.63)	< 0.001*	0.57 (0.42–0.69)	< 0.001*
Horizontal trust (TE)	0.26 (0.01–0.47)	0.034*	0.29 (0.05–0.50)	0.017*	0.33 (0.09–0.53)	0.006*	0.26 (0.01–0.47)	0.034*
Vertical trust (TM)	0.81 (0.72–0.87)	< 0.001*	0.64 (0.50–0.75)	< 0.001*	0.73 (0.61–0.81)	< 0.001*	0.64 (0.51–0.75)	< 0.001*
Organisational justice (JU)	0.82 (0.73–0.87)	< 0.001*	0.66 (0.53–0.76)	< 0.001*	0.70 (0.58–0.79)	< 0.001*	0.65 (0.52–0.76)	< 0.001*
Self‐Rated health (GH)	0.09 (−0.15–0.31)	0.455	−0.05 (−0.28–0.18)	0.644	0.03 (−0.20–0.25)	0.804	−0.18 (−0.39–0.05)	0.110

*Note:* The reported *p* values were not adjusted for multiple testing and should be interpreted as exploratory.

Abbreviations: CI, confidence interval; N/A, not applicable because the subscale is correlated with itself.

^a^ Confidence intervals were estimated using Fisher's r‐to‐z transformation (Bishara and Hittner 2017).

^b^ Standard errors were estimated using the formula proposed by Fieller, Hartley and Pearson (Bishara and Hittner 2017).

* Significant at *p* < 0.05.

### 3.4 ‘Sense of Community’ Correlations

The mean score for the ‘sense of community’ subscale was 60.04 out of 100 (SD = 29.60), indicating that participants generally reported a positive sense of belonging at work. The psychosocial factors that had strong positive correlations with the ‘sense of community at work’ subscale were: ‘social support from colleagues’ (*r* = 0.77, *p* < 0.001), ‘social support from supervisor’ (*r* = 0.70, *p* < 0.001), ‘quality of leadership’ (*r* = 0.67, *p* < 0.001), ‘organisational justice’ (*r* = 0.66, *p* < 0.001), ‘job satisfaction’ (*r* = 0.64, *p* < 0.001), ‘vertical trust’ (*r* = 0.64, *p* < 0.001) and ‘predictability’ (*r* = 0.63, *p* < 0.001) (see Table 4).

### 3.5 ‘Social Support From Supervisor’ Correlations

The mean score for the ‘social support from supervisor’ subscale was 64.77 out of 100 (SD = 28.24), indicating that participants generally reported favourable support from their supervisors. The psychosocial factors that had strong positive correlations with the ‘social support from supervisor’ subscale included: ‘social support from colleagues’ (*r* = 0.76, *p* < 0.001), ‘quality of leadership’ (*r* = 0.73, *p* < 0.001), ‘vertical trust’ (*r* = 0.73, *p* < 0.001), ‘sense of community at work’ (*r* = 0.70, *p* < 0.001), ‘organisational justice’ (*r* = 0.70, *p* < 0.001), ‘job satisfaction’ (*r* = 0.64, *p* < 0.001), ‘possibilities for development’ (*r* = 0.62, *p* < 0.001) and ‘role clarity’ (*r* = 0.62, *p* < 0.001) (see Table 4).

### 3.6 ‘Social Support From Colleagues’ Correlations

The mean score for the ‘social support from colleagues’ subscale was 61.69 out of 100 (SD = 28.80), indicating that participants generally reported favourable support from colleagues. The psychosocial factors that had strong positive correlations with the ‘social support from colleagues’ subscale were: ‘sense of community at work’ (*r* = 0.77, *p* < 0.001), ‘social support from supervisor’ (*r* = 0.76, *p* < 0.001), ‘organisational justice’ (*r* = 0.65, *p* < 0.001), ‘quality of leadership’ (*r* = 0.64, *p* < 0.001), ‘vertical trust’ (*r* = 0.64, *p* < 0.001) and ‘influence at work’ (*r* = 0.61, *p* < 0.001) (see Table 4).

## 4 Discussion

The aim of this study was to provide new insights into the experiences of sessional nursing teaching staff in Australia. Understanding participants' demographic characteristics and the factors influencing their working experiences in nurse education is important for improving working conditions, maintaining a stable workforce and supporting student outcomes. Although participants generally reported favourable working experiences, these findings contrast with literature describing the precarious conditions associated with sessional academic employment and therefore require cautious interpretation.

Internationally, sessional staff employed by universities are often industry experts nearing the end of their careers or already retired (Clarke 2021; McComb and Eather 2024). This trend is seen in many disciplines including accounting, science, education and engineering (Crimmins et al. 2017; Khosa et al. 2025; Smith and Urquhart 2018). The findings of this study reflect a similar pattern, with over 51% of participants aged above 50. In contrast, only 30% of the currently registered nursing workforce in Australia falls into this group (Nursing and Midwifery Board of Australia 2024), highlighting a notable age difference that warrants consideration when managing the sessional nursing workforce.

Nurses appear to be moving into academia later in their careers, often bringing extensive clinical experience. In this study, 89.2% of participants had more than 10 years of clinical practice experience (*n* = 75). This may be due to a desire to give back to the nursing profession after establishing their clinical career, or due to burnout and stress from clinical nursing practice or not enjoying clinical work (Bodak et al. 2019; Dixon et al. 2015; Raso et al. 2021). Sessional nursing academics bring extensive clinical knowledge and experience to nursing education, enabling them to bridge theory and practice and contextualise real‐world workplace issues for students (Clarke 2021; Ricks et al. 2025). Universities may strategically leverage this expertise to attract nursing professionals into educational roles, thereby enhancing the quality and relevance of their nursing programmes. Conversely, some permanent academics transitioning to retirement may move to sessional work for supplemental income or reduced responsibilities, often preferring to focus on teaching and marking (East et al. 2024; McComb and Eather 2024). It is essential that academic leadership teams provide appropriate support to meet the diverse needs of sessional academics.

Flexibility appeared to be an important reason for working as a sessional teaching staff member. Given that 71.4% of participants reported carer responsibilities, the flexibility of sessional work may be associated with their ability to balance teaching with family, caring, clinical or personal commitments. Sessional work may also be undertaken remotely for activities such as online facilitation, lecturing and marking (Ndzinisa and Dlamini 2022). Remote sessional work may allow staff to continue using their nursing knowledge and skills without always being required to attend campus and may contribute to retaining nurses within the discipline (Bodak et al. 2019). Flexible employment arrangements can be an important motivator for sessional staff because they may support job satisfaction and contribute to workforce sustainability (Fine et al. 2019; Jessiman‐Perreault et al. 2024; Jiang et al. 2023).

### 4.1 Sessional Teaching Career Aspiration

Most participants did not indicate a definite intention to become permanent academics, with only 26.2% (*n* = 22) reporting that they intended to pursue a permanent role. This pattern may reflect both positive preferences for sessional employment and structural barriers to permanent academic work. Some sessional staff may value the flexibility to combine teaching with clinical practice, caring responsibilities or other personal commitments. In contrast, permanent academic roles may involve broader teaching, research, administrative and service expectations, greater workload and reduced flexibility, while some sessional staff may not yet meet the qualifications or experience requirements for permanent employment (Bodak et al. 2019; East et al. 2024; Ricks et al. 2025). For some participants, sessional work may therefore provide a pathway to permanent employment, whereas for others it may be a preferred form of bounded academic engagement.

In this study, 58.3% of participants had less than 5 years of sessional academic experience (*n* = 49), indicating that a substantial proportion of the sample was relatively new to sessional academic work. The casual nature of sessional employment may reduce continuity within teaching teams, which may have implications for consistency in teaching delivery. From a workforce planning perspective, universities may need to provide repeated onboarding and role preparation for sessional staff as teaching teams change across semesters, which can increase administrative workload (Brouwer et al. 2024). Universities may need to consider different ways to manage and support sessional nursing staff such as providing financial or professional development opportunities to support their career growth in nurse education. High turnover and limited continuity among sessional staff may disrupt the consistency of teaching, assessment practice and student‐educator relationships within nursing programmes. Such discontinuity may affect students' learning experiences by reducing opportunities for sustained academic support and mentorship, which are important for developing clinical reasoning and professional identity in nursing education (Dayagbil et al. 2021).

Sessional staff can be motivated by the job itself including having the ability to teach nursing students and influence the next generation of the nursing workforce (Bodak et al. 2019; Xie et al. 2023). Several studies have found that sessional staff feel that they play an important role in giving back to the nursing profession and have the ability to influence and nurture the next generation of the nursing workforce (Dixon et al. 2015; East et al. 2024; Evans 2018; Ricks et al. 2025; Woodworth 2017). This was demonstrated by the ‘meaning of work’ subscale which positively correlated with the ‘job satisfaction’ subscale. Participants reported relatively low levels of illegitimate tasks and emotional demands. These findings may reflect the allocation of clearly defined teaching and marking duties to sessional staff rather than an overall reduction in the workload required to deliver nursing programmes. The ad hoc nature of sessional work means staff are paid for the tasks required by the program such as teaching or marking (Brouwer et al. 2024). However, ongoing academic, administrative and coordination responsibilities may remain with permanent academics. Reliance on sessional staffing may therefore redistribute workload across the academic team, highlighting the need for a balanced staffing model that considers the workload and sustainability of both sessional and permanent academics. A weak negative correlation was observed between ‘job satisfaction’ and ‘illegitimate tasks’ (*r* = −0.21, *p* = 0.069); however, this association was not statistically significant. Therefore, this study did not provide sufficient quantitative evidence to conclude that illegitimate tasks were associated with job satisfaction among sessional nursing academics. Nevertheless, the low mean score for ‘illegitimate tasks’ suggests that participants generally did not report high levels of unnecessary or inappropriate tasks in their sessional roles.

### 4.2 Working Conditions

Participants' ‘job satisfaction’ was strongly associated with ‘role clarity’, ‘predictability’, ‘quality of leadership’ and ‘possibilities for development’. These associations suggest that satisfaction may be related to how sessional work is organised and supported, including the provision of clear expectations, timely communication, effective leadership and development opportunities. However, favourable perceptions of these day‐to‐day organisational conditions do not remove the structural insecurity associated with sessional employment and should therefore be interpreted cautiously. Sessional staff felt that they had a positive working environment when there were clear role boundaries and descriptions including informed teaching plans, notification of changes, decisions about curriculum and resources in advance and good quality leadership support to function (Bodak et al. 2019; Christian et al. 2021; East et al. 2024). Setting clear boundaries allowed sessional staff to have less cognitive workload and focus on the required tasks efficiently (Hitch et al. 2018; Tamimi and Tamam 2023). The positive mean value of the ‘predictability’ subscale demonstrates that sessional staff had adequate information to do their job and were informed of any changes or plans about their roles well in advance (Heffernan 2018; Patterson et al. 2022; Ricks et al. 2025). This may also contribute to the positive outcome of the ‘role conflict’ subscale as sessional staff may experience fewer competing role expectations than permanent academics. In addition, workplace stability and effective quality leadership and management can improve the sessional staff's working experience (Papa et al. 2020; Yadav 2020). This may be influenced by orientation and the onboarding process which should clearly outline the expectations of the university to provide clarity in sessional staff roles and functions (Brouwer et al. 2024, 2026). However, these favourable perceptions may reflect the institutional contexts represented in this sample and the greater likelihood of participation by sessional academics with more positive working experiences. Therefore, the findings should not be generalised to all sessional nursing academics.

Sessional staff generally come from clinical backgrounds and may have limited formal preparation in teaching and assessment of nursing students (Garvey et al. 2025; Ricks et al. 2025). International literature suggests that sessional staff can be better supported through access to professional development, career growth opportunities and ongoing institutional support (Harvey 2017; Hitch et al. 2018; McComb et al. 2021; McComb and Eather 2024). Universities can support sessional staff by providing professional development in teaching pedagogy and educational practice. Such support may strengthen sessional staff's confidence and capability in teaching roles, particularly when they understand the rationale underpinning educational practices (Clarke 2021; Hollar et al. 2022; Judge et al. 2020; Khosa et al. 2025; McComb and Eather 2024; Siddiqui 2019; Tower et al. 2024). Adequate educational support may also increase motivation, productivity and the quality of teaching and student learning experiences (Arian et al. 2018).

### 4.3 Security and Community

Sessional work is often characterised by short‐notice hiring, hourly payment and limited employment security (Brouwer et al. 2024). This would usually pose a high level of job insecurity (McComb and Eather 2024; Skead and Rogers 2023); however, participants reported relatively low perceived job insecurity and low insecurity over working conditions, as indicated by the low mean scores for these subscales. This suggests that although sessional employment remains structurally insecure, participants in this study did not perceive their employment situation as highly unstable or threatening. Many sessional staff appeared to feel confident about the continuation of their roles due to ongoing teaching demand and repeated engagement across the semester which may contribute to a sense of predictability in their working conditions. Furthermore, the low mean for the ‘Work Life Conflict’ subscale suggested that sessional staff felt they had a good level of work‐life balance in their roles. This suggests that the flexibility associated with sessional teaching may allow staff to manage their professional and personal commitments without experiencing significant conflict between work and personal life.

A positive working experience can be fostered by having a strong sense of community which can be promoted through good support from supervisors and colleagues for sessional staff (Ricks et al. 2025). Our findings show that sessional staff reported having a positive ‘sense of community’, meaning they generally felt they were part of the nursing teaching team and they had a good relationship with their colleagues. Sessional staff reported they could get help if needed from both their supervisors and colleagues and that this contributed to improving the sense of belonging. Adequate feedback and support can lead sessional staff to perceive they belong to the teaching team, improving their performance and further contributing to an enjoyable workplace (Bodak et al. 2019; Ricks et al. 2025).

### 4.4 Vertical Trust and Organisational Justice

A trusting relationship between sessional staff and the leadership team facilitates conflict resolution and promotes a positive working environment (Dahleez and Aboramadan 2022; McNaughtan et al. 2022). We found participants had positive ‘vertical trust’ and ‘organisational justice’, meaning sessional staff believed the leadership and management team trusted them to do their work well and that conflicts were resolved fairly. This also demonstrates that sessional staff appreciated having autonomy in their work including the ability to express themselves. Trust in employees has been found to increase productivity, retention and job satisfaction (Jain 2016; Rakhra 2018). This is demonstrated when the management team provides a good level of autonomy for sessional staff to complete their assigned tasks such as marking and teaching without being micromanaged; however, this requires a high level of role clarity, expectations and support (Singh et al. 2019; Tamimi and Tamam 2023).

## 5 Limitations

Participants in this study were from Australian universities which may not be representative of the sessional nursing staff experience worldwide. Future studies may consider recruiting participants globally. Although participants represented 25 educational institutions and varied in their demographic and professional characteristics, no national sampling frame or reliable demographic profile of the Australian sessional nursing academic workforce was available. Therefore, it was not possible to determine whether the sample was representative of the broader Australian sessional nursing academic workforce. The use of convenience sampling and voluntary participation may also have introduced self‐selection bias, as sessional nursing academics who were more engaged with or interested in their working experiences may have been more likely to participate. Sessional nursing academics with more stable or positive employment experiences may have been overrepresented, which may partly explain the relatively low perceived job insecurity and favourable working‐condition findings. As this study used a cross‐sectional design, the reported correlations indicate associations and cannot establish causal relationships between workplace factors and participants' experiences. The use of self‐reported survey data may also have introduced response bias including differences in how participants interpreted and reported their working experiences.

We did not observe any negative correlations between ‘job satisfaction’, ‘sense of community’, ‘social support from supervisor’, ‘social support from colleagues’ and other subscales. The absence of negative correlations may also reflect this potential participation bias. Only the middle‐length version of the COPSOQ–III instrument was used whereas other psychosocial factors related to work experience in the long version including stress, burnout, violence and harassment were not collected from participants as they are hypothesised to have negative correlations with job satisfaction and the sense of community. These would be valuable insights to better understand sessional staff's work experience in nurse education. Although this study provides a valuable perspective on sessional nursing staff's job satisfaction and the sense of community, the reasons for their choices are not well understood. The nuances of sessional staff experiences could be better understood through qualitative or mixed‐method studies.

## 6 Conclusion

This study presented a demographic profile of sessional nursing staff at Australian universities. The profile showed substantial variation in participants' age and years of sessional academic experience, suggesting that management and support practices may need to respond to differing levels of academic experience and professional circumstances within the sessional nursing academic workforce. Sessional staff brought strong clinical experience to their teaching roles and provided value to nursing students' learning by linking clinical knowledge with nursing education. Universities should continue to engage nursing experts in the nursing curriculum within a balanced workforce model that strengthens organisational support for sessional staff while ensuring that teaching, administrative and coordination responsibilities are distributed sustainably across sessional and permanent academics. Participants generally reported favourable working experiences, including trusting workplace relationships, opportunities to influence the future nursing workforce and the ability to contribute to the nursing profession through sessional teaching. Universities can further strengthen factors correlated with job satisfaction by providing professional development opportunities that enhance sessional staff's educational capabilities and career development. These strategies may improve sessional staff's working experiences and contribute to the sustainability of the nurse education workforce.

## Author Contributions

**Thomas Aaron Ricks:** conceptualization, methodology, data curation, investigation, formal analysis, project administration, writing – original draft, writing – review and editing. **Elisabeth Jacob:** conceptualization, methodology, validation, supervision, writing – review and editing. **Louisa Lam:** conceptualization, methodology, supervision, writing – review and editing, validation.

## Funding

The authors have nothing to report.

## Conflicts of Interest

The authors declare no conflicts of interest.

## Supporting information


**File S1:** Internal consistency of COPSOQ–III.

## Data Availability

The data that support the findings of this study are available on request from the corresponding author. The data are not publicly available due to privacy or ethical restrictions.
