# Supplementary material for: Work Experience of Sessional Teaching Staff in Pre‐Registration Nursing Programmes: A Cross‐Sectional Study
Source: Nurs Open. 2026 Sep 29;13(10):e70862. doi: 10.1002/nop2.70862 (PMC13621412; doi:10.1002/nop2.70862)
Supplement: Supplementary file 1 [file NOP2-13-e70862-s001.docx]

**Supplementary File 1:** Internal consistency of COPSOQ–III

| **Subscale and abbreviation** | **Cronbach’s alpha** |
| --- | --- |
| Quantitative Demands (QD)^a^ | 0.564 |
| Work Pace (WP)^a,b^ | 0.285* |
| Emotional Demands (ED)^a,b^ | 0.777 |
| Demands for Hiding Emotions (HE)^a,b^ | 0.714 |
| Influence at Work (IN)^a^ | 0.710 |
| Possibilities for Development (PD)^b^ | 0.637 |
| Control over Working Time (CT)^a^ | 0.814 |
| Meaning of Work (MW)^b^ | 0.867 |
| Predictability (PR)^b^ | 0.692 |
| Recognition (RE)^b^ | N/A |
| Role Clarity (CL)^b^ | 0.805 |
| Role Conflicts (CO)^b^ | 0.750 |
| Illegitimate Tasks (IT)^b^ | N/A |
| Quality of Leadership (QL)^b^ | 0.813 |
| Social Support from Supervisor (SS)^a^ | 0.656 |
| Social Support from Colleagues (SC)^a^ | 0.829 |
| Sense of Community at Work (SW)^a^ | 0.829 |
| Job Insecurity (JI)^b^ | 0.805 |
| Insecurity over Working Conditions (IW)^b^ | 0.723 |
| Quality of Work (QW)^b^ | N/A |
| Job Satisfaction (JS)^c^ | 0.664 |
| Work Life Conflict (WF)^a^ | 0.922 |
| Horizontal Trust (TE)^b^ | N/A |
| Vertical Trust (TM)^b^ | 0.891 |
| Organizational Justice (JU)^b^ | 0.706 |
| Self Rated Health (GH)^d^ | N/A |
| N/A = Not applicable as subscale contains one item.  *The Work Pace subscale demonstrated low internal consistency and was therefore excluded from further analysis.  Likert scales:  a. Always (100); Often (75); Sometimes (50); Seldom (25); Never/hardly ever (0), Missing/No supervisors/colleagues (coded as missing).  b. To a very large extent (100); To a large extent (75); Somewhat (50); To a small extent (25); To a very small extent (0).  c. Very satisfied (100); Satisfied (75); Neither/Nor (50); Unsatisfied (25); Very unsatisfied (0).  d. Excellent (100); Very good (75); Good (50); Fair (25); Poor (0). | |
